# Genetics of cognitive trajectory in Brazilians: 15 years of follow-up from the Bambuí-Epigen Cohort Study of Aging

**DOI:** 10.1038/s41598-019-53988-4

**Published:** 2019-12-02

**Authors:** Mateus H. Gouveia, Cibele C. Cesar, Meddly L. Santolalla, Hanaisa P. Sant Anna, Marilia O. Scliar, Thiago P. Leal, Nathalia M. Araújo, Giordano B. Soares-Souza, Wagner C. S. Magalhães, Ignacio F. Mata, Cleusa P. Ferri, Erico Castro-Costa, Sam M. Mbulaiteye, Sarah A. Tishkoff, Daniel Shriner, Charles N. Rotimi, Eduardo Tarazona-Santos, Maria Fernanda Lima-Costa

**Affiliations:** 1 Fundação Oswaldo Cruz, Instituto de Pesquisas René Rachou, Belo Horizonte, Brazil; 2 Departamento de Genética, Ecologia e Evolução, Instituto de Ciências Biológicas, Universidade Federal de Minas Gerais, Belo Horizonte, MG 31270-901 Brazil; 3 Center for Research on Genomics and Global Health, National Human Genome Research Institute, National Institutes of Health, Bethesda, Maryland United States of America; 4 Universidade Federal de Minas Gerais, Faculdade de Ciências Econômicas, Belo Horizonte, Brazil; 5 Melbourne Integrative Genomics, The University of Melbourne, Melbourne, VIC 3052 Australia; 6 Núcleo de Ensino e Pesquisa - NEP, Instituto Mário Penna, Rua Gentios, Terceiro Andar, Belo Horizonte, Minas Gerais 3052 Brazil; 7 Lerner Research Institute, Genomic Medicine, Cleveland Clinic Foundation, Cleveland, OH USA; 8 Universidade Federal de São Paulo, Department of Psychiatry, São Paulo, Brazil; 9 Division of Cancer Epidemiology and Genetics, National Cancer Institute, National Institutes of Health, Bethesda, Maryland United States of America; 10 Department of Genetics, University of Pennsylvania, Philadelphia, Pennsylvania United States of America

**Keywords:** Genome-wide association studies, Rare variants

## Abstract

Age-related cognitive decline (ACD) is the gradual process of decreasing of cognitive function over age. Most genetic risk factors for ACD have been identified in European populations and there are no reports in admixed Latin American individuals. We performed admixture mapping, genome-wide association analysis (GWAS), and fine-mapping to examine genetic factors associated with 15-year cognitive trajectory in 1,407 Brazilian older adults, comprising 14,956 Mini-Mental State Examination measures. Participants were enrolled as part of the Bambuí-Epigen Cohort Study of Aging. Our admixture mapping analysis identified a genomic region (3p24.2) in which increased Native American ancestry was significantly associated with faster ACD. Fine-mapping of this region identified a single nucleotide polymorphism (SNP) rs142380904 (β = −0.044, SE = 0.01, *p* = 7.5 × 10^−5^) associated with ACD. In addition, our GWAS identified 24 associated SNPs, most in genes previously reported to influence cognitive function. The top six associated SNPs accounted for 18.5% of the ACD variance in our data. Furthermore, our longitudinal study replicated previous GWAS hits for cognitive decline and Alzheimer’s disease. Our 15-year longitudinal study identified both ancestry-specific and cosmopolitan genetic variants associated with ACD in Brazilians, highlighting the need for more trans-ancestry genomic studies, especially in underrepresented ethnic groups.

## Introduction

Age-related cognitive decline (ACD) is the gradual and enduring decline of cognitive function with increasing age^1^. ACD progression is likely due to the combined effects of aging process and underlying chronic conditions such as type 2 diabetes (T2D), cerebrovascular, and inflammatory diseases^2–5^. Latin American populations have some of the highest prevalence rates of dementia in the world^6^, highly increased with age^7^. Although ACD is not only observed in people with dementia, identifying factors associated with changes in cognitive function is fundamental to the understanding of dementia.

The contributing influence of genetic factors to cognitive decline is receiving increasing attention. Single nucleotide polymorphisms (SNPs) within several genes, including *APOE*, *TOMM40*, and insulin resistance-associated genes, have been associated with ACD^1^. A collaborative analysis of 14 cohort studies from 12 countries showed a robust association between ACD and the *APOE* ε4 allele carrier status^8^. However, the influence of genomic ancestry on ACD is controversial. A recent admixture mapping and meta-analysis of five African American cohorts reported that SNPs associated with susceptibility to Alzheimer’s disease in the *ABCA7* and *MS4* loci are also associated with ACD^9^. Although this study did not report ancestry-related genomic regions significantly associated with ACD, it noted faster cognitive decline in individuals with a higher genome-wide proportion of African ancestry. In contrast, in the single study in older Latin American adults from the Bambuí-Epigen Cohort Study of Aging, we showed that higher genome-wide proportion of African ancestry was associated with lower cognitive function at baseline but not with the trajectory of cognitive function and hence not with ACD^10^. Additionally, the association of African ancestry with baseline cognitive function was modified by educational level^10^. Moreover, we did not find association between the genome-wide proportion of Native American ancestry and either baseline cognitive function or its trajectory^10^.

Considering Latin American populations are products of a complex pattern of admixture of African, European, and Native American ancestral sources^11,12^, they are suitable populations for admixture mapping analysis of the trajectory of cognitive function. Admixture mapping uses an admixed population to search for ancestry-related genomic regions associated with phenotypes^13^. To date, in Latin America, there are no reports of either admixture mapping or genome-wide association studies (GWAS) investigating the association between ancestry-related genomic regions or genetic markers with the trajectory of cognitive function.

To examine the influence of ancestry-related genomic regions as well as single genetic variants on the trajectory of cognitive function in Brazilians, we performed admixture mapping followed by fine-mapping and genome-wide association study (GWAS) using data from 15 years of cognitive function assessment and ~2.5 million SNPs genotyped in 1,407 individuals from the Bambuí-Epigen Cohort Study of Aging^14^.

## Results

### Population and age-related cognitive decline (ACD)

**Figure 1:**
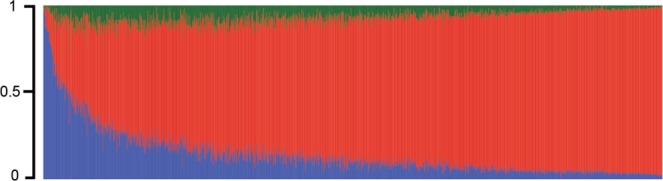
Tri‐hybrid genome‐wide individual proportion of ancestry of 1,407 participants of the Bambuí‐Epigen Cohort Study of Aging. Each bar represents one individual and the green, red, and blue colors in the bar plot represent the Native American, European, African ancestry, respectively.

Among 1,407 study participants, the mean age was 69 years (Standard deviation [SD] = 7.1); female gender (61%) and very low schooling level predominated (64.2% had less than 4 years, 27.9% had 4–7 years and 7.8% had 8 years or more of formal education). The global median European, African and Native American Ancestry was 83.8% (Interquartile range [IRQ] = 17.4%), 9.6% (IQR = 12.8%) and 5.4% (IQR = 5.6%), respectively (Fig. 1). This ancestry profile is similar to other Brazilians from Southeast/South of the country^11,15,16^. The baseline average Mini-Mental State Examination (MMSE) score was 28 (SD = 4.2). Over 15 years of follow-up, 37% individuals died, 6% were lost for follow-up, and 14,956 MMSE measures were made, which were used to obtain the per-individual slope of the cognitive trajectory (see Methods for details) that we call “age-related cognitive decline” (ACD). The median number of MMSE measures was 11 per individual, with first and third quartiles equal to 6 and 15 measures, respectively. After mixed model analysis, 76% of the regression coefficients were negative (meaning decline of cognitive trajectory), 23.8% were positive (meaning increasing) and 0.2% were null, meaning flat trajectory.

### Admixture mapping and fine-mapping

**Figure 2:**
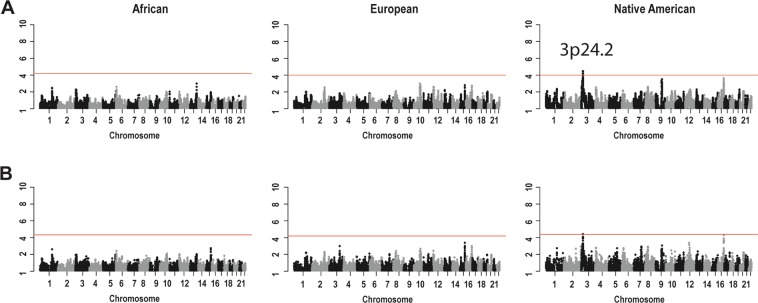
Admixture mapping scans of African, European and Native American genomic segments of ancestry. The Admixture mapping analyses were performed using both (**A**) RFMix and (**B**) PCAdmix methods to infer the local ancestry. The horizontal red line represents the significance threshold for each admixture mapping test (see methods).

To determine whether local ancestry is associated with age-related cognitive decline (ACD), we performed admixture mapping analyses in Brazilians assuming African, European, and Native American ancestral sources. Using local ancestry estimates from both RFMix and PCAdmix methods, our admixture mapping analyses detected a genomic region (3p24.2) at which increased Native American ancestry was associated with ACD (β* = *−0.022, SE = 0.005, *p* = 3.9 × 10^−5^), but not with either African or European ancestries (Fig. 2A,B). We further performed an admixture mapping “extreme outcome” strategy^9^. It was comprised of the subsetting of the highest and lowest 20% (n = 564) individuals from the ACD distribution (defined as cases and controls, respectively), and then, a case-control admixture mapping analysis was performed. Using this approach, we confirmed the admixture mapping association in the region 3p24.2 (β* = *−0.20, SE = 0.056, *p* = 4 × 10^−4^), and again we did not observe any significant admixture mapping associations for African or European ancestries. Despite this, we found a slightly higher proportion of global European ancestry in extreme cases (83% ± 13%) compared with controls (77% ± 18%) (*p* = 1.6 × 10^−4^).

**Figure 3:**
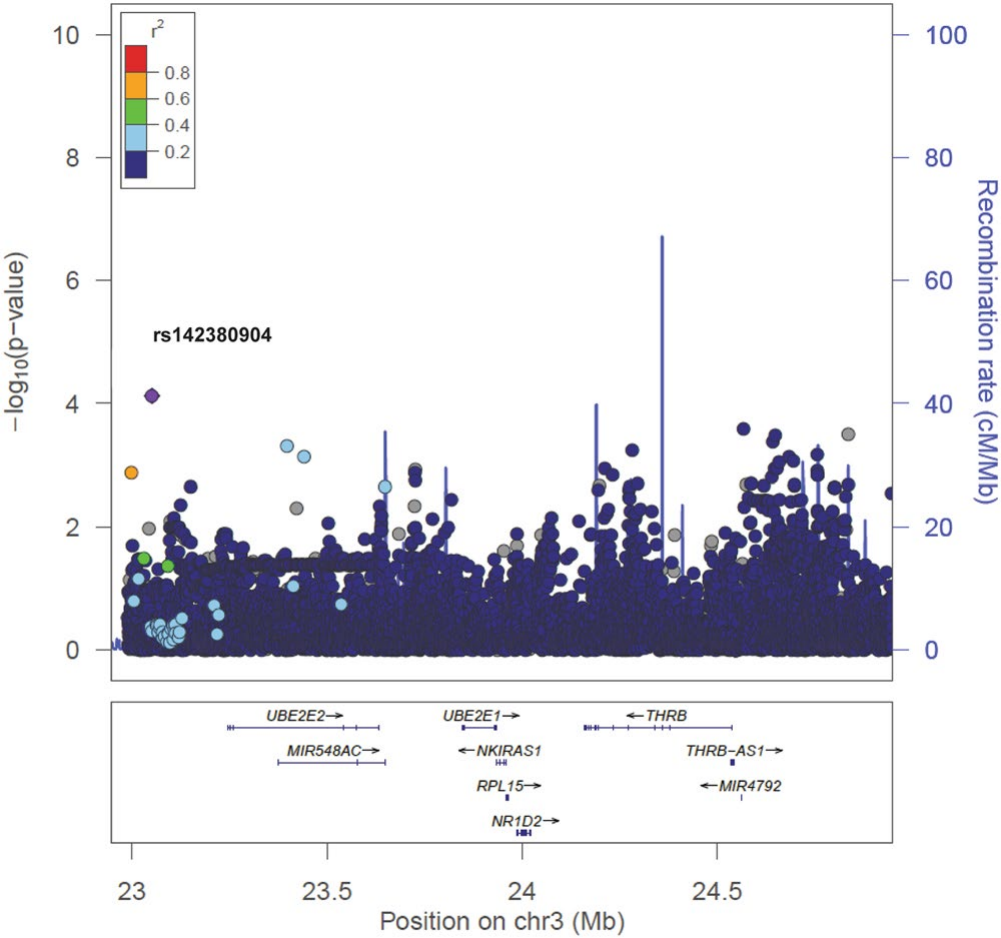
LocusZoom plot of fine mapping of the (3p24.2) region using both genotyped and imputed SNPs ± 1 Mb from the target region. The SNP rs142380904 with the lowest p-value is color coded in purple and labeled. The linkage disequilibrium between this SNP and the remaining nearby SNPs is indicated by the color coding according to *r*^2^ values based on admixed Americans (AMR) from 1000 Genomes Project.

Next, we performed fine-mapping analysis in the region of the significant admixture mapping peak at 3p24.2 (Fig. 3). We identified a significant association with ACD for rs142380904 (β* = *−0.044, SE = 0.01, *p* = 7.5 × 10^−5^), which is located in a region previously associated with atypical psychosis^17^. Also, the fine-mapped region covers genetic markers and genes associated with type 2 diabetes mellitus, colorectal cancer, red blood cell, and hemoglobin traits (Fig. 3). At rs142380904, the derived allele T is associated with ACD and is rare to absent in European (0.2%), African (0%), and East Asian (0%) populations but has a frequency of 13.1% in admixed American populations from Colombia, Mexico, Peru, and Puerto Rico^18^. The highest frequency (30.6%) of the T allele was found in Peruvians from Lima (PEL)^18^, who have an average of more than 80% of Native American ancestry^19^. Despite the rs142380904-T allele showing a low frequency (1.5%) in our Brazilian study population, 43 carriers showed a decline in age-related cognitive function 27 times faster than those without the T allele (1,364 homozygous individuals, *p* = 4.8 × 10^-6^). Using HaploReg v4.1^20^, we identified two variants in linkage disequilibrium (LD) with rs142380904 in the 1000 Genomes Native American ancestry (AMR) samples: rs5847212 (r^2^ = 0.82) and rs139451855 (r^2^ = 0.61). Notably, rs5847212 is a significant eQTL of the gene *NR1H2*, which has been shown to have an important role in the development of Alzheimer’s disease-related pathology^21^. Furthermore, both variants (rs5847212 and rs139451855) are ~190 to 250 kb upstream of the gene *UBE2E2*. UBE2E2 interacts with Siah proteins, which regulate synaptophysin degradation; synaptophysin has been associated with long-term potentiation, neurotransmitter release, and neurodegenerative diseases including Alzheimer disease^22^. According to Genotype-Tissue Expression (GTEx) data (https://www.gtexportal.org/home/), *UBE2E2* is ubiquitously expressed, including all sampled regions of the brain.

### Genome-wide association analysis

**Figure 4:**
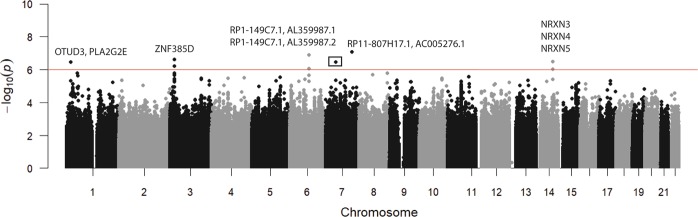
Manhattan plot of the genome-wide association study (GWAS) analysis. The plot represents the −log10 transformed p values for all 10.4 M SNPs analyzed. The horizontal red line represents the standard genome-wide suggestive significance (*p* = 10^-6^). The rectangle highlights the hits for the five SNPs in the chromosome 7 (Table S1).

Although we found one ancestry-related genomic region associated with ACD, several genetic variants are reported to influence this trait^1^. To search for genome-wide variants associated with ACD in our Brazilian study population, we performed a GWAS using genotyped and imputed SNPs (Fig. 4). We identified 24 SNPs suggestively (*p* ≤ 10^−7^) associated with the trajectory of cognitive function (Fig. 4, Table S1). Of these 24 SNPs, 12 are closely located in the gene *ZNF385D*. We also identified three peaks (*p* ≤ 10^−7^) for SNPs within three different neurexin family genes (*NRXN3-5*), which function in the nervous system as receptors and in cell adhesion. We detected nine additional hits in intergenic regions that were previously not known to be associated with ACD (Fig. 4 and Table S1).

We then evaluated selected GWAS Catalog SNPs (Welter *et al*., 2014) previously associated with “cognitive decline” and/or “Alzheimer’s disease” (reference file “All associations” v1.0). We identified association (*p* < 0.05) between ACD and the risk allele of 26 of 468 previously reported SNPs (Table S2). Most of these genetic markers were related to lipid metabolism and the strongest association (*p* = 6 × 10^−5^) was identified for the SNP rs429358 in *APOE*.

### Significant SNPs and variance explained

As described above, we identified a total of 25 SNPs associated (one using admixture mapping and 24 using GWAS) with ACD using the combined approaches of admixture mapping, fine-mapping, and GWAS. From these SNPs, we used a stepwise model selection procedure to select the top SNPs: rs138347004, rs76238412, rs142380904, rs190722109, rs78400729, and rs77499314. The set of six SNPs explained 18.5% (SE = 0.082, *p* = 0.015) of the proportion of phenotypic variance.

## Discussion

Our approach, which includes admixture-mapping, fine-mapping and genome-wide association study (GWAS), revealed a genomic region enriched for Native American ancestry as well as single SNPs associated with age-related cognitive decline (ACD) in the Bambuí-Epigen Cohort Study of Aging. Because the study had a longitudinal design, our associations analyses allows the identification of genetic determinants of cognitive function over time.

Previously, we did not find any association between the global African or Native American ancestries and the trajectory of cognitive function in the Bambuí cohort population (Lima-Costa *et al*. 2018). The current admixture mapping analysis (based on local ancestry) provides more evidence that African ancestry is not associated with cognitive trajectory in this admixed population. In contrast, an excess of Native American ancestry at chromosome region 3p24.2 emerged as being associated with a more negative trajectory of cognitive function, or faster cognitive decline (Fig. 2A,B). Subsequent fine-mapping of this region highlighted the SNP rs142380904. At this SNP, the allele that was associated with faster cognitive decline has its highest frequency in the Peruvian population from Lima, Peru (PEL), which has the highest proportion of Native American ancestry among the 1000 Genomes Project populations^18^. We did not find any regions enriched for African or European ancestry associated with ACD, in agreement with the single previous admixture mapping of ACD using an African American cohort (Raj *et al*., 2017). There is no previous admixture mapping of ACD using Native American-ancestry populations or available data to replicate our Native American-associated region. However, the candidate positional gene *UBE2E2*, which encodes an E2 ubiquitin-conjugating enzyme, has been associated with atypical psychosis and type 2 diabetes^22^, in addition to neurotransmitter release and neurodegenerative disease, consistent with ubiquitous tissue expression and pleiotropy. Only 7% of carriers of the rs142380904-T allele had type 2 diabetes (T2D, as measured by HbA1c serum levels and/or treatment), while 15% of non-carriers had T2D. Therefore, it’s unlikely that T2D mediates the association between UBE2E2 and cognitive trajectory in our data.

Even with our limited sample size, our GWAS analysis identified SNPs within genes previously reported to be related via functional analysis and/or association studies to cognitive function. Most of the associated SNPs are within *ZNF385D*, which was previously reported to be associated with reading disability and language impairment^23^. Additionally, we found associated SNPs within three neurexin family genes. Notably, *NRXN3* is associated with autism spectrum disorder^24^ and borderline personality disorder^25^. Also, *NRXN4* (*CNTNAP1*) has been identified in abnormal axon myelination^26^.

The top SNPs we identified to be associated with ACD, combined across admixture mapping and GWAS, explained 18.5% of the phenotypic variance. In addition, we replicated some markers previously associated with cognitive decline and/or Alzheimer’s disease. Taken together, these results suggest that there are several markers displaying genetic contributions to cognitive decline. This observation agrees with the consensus report that the heritability of behavior-related genes is attributable to many variants with small effects^27^.

This study has strengths and limitations. Strengths include a large community-based sample of an admixed population, followed over a very long period with annual measures of cognitive function. Also, there was minimal loss of participants during follow-up, resulting in an average of 11 MMSE measures per participant. A limitation of our study is inherent to longitudinal studies of aging in which older adults are at greater risk of death, which in turn might lead to differential censoring. To address this survival bias, we considered dropouts in our mixed models.

We adjusted our analysis for age, sex and educational level, which have been previously described as the most important factors in explaining cognitive trajectory^8–10^. In a sensitive analysis we further adjusted our model for lifestyle and health conditions following Lima-Costa *et al*.^10^, we did not find differences in the cognitive trajectories estimated by those models.

A limitation in our analysis is the absence of measure of dementia, which is a difficult task in large population-based studies. As in other large population-based studies^8,9^, we assume here that the rate of cognitive decline is a reasonable proxy for dementia incidence, as we expect that more rapid cognitive decline will lead to higher rates of dementia^8,9^. An advantage on the use of cognitive trajectory includes the measurement of cognitive changes over time and the examination of individual-specific patterns of decline. Finally, it is important to note that, although the MMSE is among the most widely-used tests of cognitive function in old age, it is a global measurement and its total score does not precisely correspond to specific cognitive domains. It is important that future analyses use a more detailed assessment of cognitive function that might reveal different patterns of association across cognitive domains.

## Conclusions

We carried out the first admixture mapping and GWAS in Latin America to determine relationships between genetic factors and the trajectory of cognitive decline. We found a genomic region at which increased Native American ancestry is associated with faster age-related cognitive decline, highlighting the need to increase the availability of Native American-ancestry datasets. Furthermore, our GWAS detected new SNP associations and replicated other SNPs associated with cognition-related outcomes. Our results lay a foundation to develop a polygenic risk score that can be used as a prognostic marker of cognitive disorders, with the potential to be used for prevention.

## Methods

### The Brazilian study population

The Bambuí-Epigen Cohort Study of Aging is ongoing in Bambuí, a city of approximately 15,000 inhabitants, in Minas Gerais State in southeast Brazil. The population eligible for the cohort comprised all residents aged ≥60 years on 1 January 1997, totaling 1,742 eligible residents. From those eligible residents, from 1997 to 2007, during a mean follow-up of 8.6 years, 641 participants died and 96 (6.0%) were lost to follow-up. The characteristics of the study participants in the baseline are described in Lima-Costa *et al*.^10^ and further details on the Bambuí cohort are described elsewhere^14^.

### Assessing age-related cognitive function

To assess cognitive function, we used the Mini-Mental State Examination (MMSE), a quantitative measure extensively used in clinical practice and research for screening cognitive function impairment^28^. The MMSE includes tests of attention, language, memory, orientation, and visual-spatial skills. The MMSE is evaluated using a score ranging from 0 to 30. Low measures may indicate cognitive impairment and decreases in measures over time may indicate cognitive decline.

The MMSE measures were obtained annually from 1997 to 2011, using a previously validated version of the MMSE^29,30^. For those individuals with at least two MMSEs measures during the 15 years of follow-up and genotype data (1,407), we used the linear mixed models function implemented in the R package lme4^31^ to fit and assess the trajectories of the cognitive function for each individual using their annual measures of MMSE, adjusting for deaths during the follow-up period and family structure. Then, we extracted the per-individual age-related cognitive decline (ACD), which is the slope of the trajectory of cognitive function over time. We adjusted for family structure because 620 study participants were previously inferred as first- or second-degree relatives and some were within family networks up to 50 individuals^11^. The exclusion of these related individuals might lead to loss of power and selection bias; therefore, we used all relatives in the analysis, correcting for family structure in all models using robust variance estimators^31^. Further details can be seen elsewhere^10^.

### Genotyping, quality control, and imputation

We genotyped 1,442 cohort participants^14^ in the context of the EPIGEN project^11^. Cohort participants were genotyped using the Illumina Omni 2.5 M array (San Diego, California). Data cleaning was performed as detailed previously^11^. We performed imputation using IMPUTE2^32^ and the 1000 Genomes Project Phase III reference panel^19^. As a quality control measure, only markers with an info score of >0.8 were considered for fine-mapping and genome-wide association analysis. Also, we excluded SNPs with minor allele frequency <0.01. All remaining genotyped and imputed SNPs were tested for association with the trajectory of cognitive function.

### Global ancestry and relatedness

We used ADMIXTURE^33^ and 370,539 SNPs to estimate global ancestry assuming three ancestral sources, using African, European, and Native American populations from public datasets as parental sources^11^.

Using the genome-wide data, we previously estimated that cohort participants have a high level of family structure^11,16^. Therefore, we inferred a matrix of kinship coefficients, taking into account global ancestry, using REAP^34^, which was developed especially for admixed populations.

### Phasing and local ancestry

We phased our genome-wide genotype data using the software SHAPEIT2^35^ as detailed previously^11^. To increase the confidence of our local ancestry estimates, we inferred local ancestry using both RFMix^36^ and PCAdmix^37^. For the ancestral populations in RFMix, we used data from the 1000 Genomes Project and data genotyped using Illumina Omni 2.5 M or 5 M arrays. To represent African ancestry, we used data from the GWD sample from the 1000 Genomes Project^19^ in addition to data from Africans from Botswana^38^ and Kwa/Gur (from the National Cancer Institute (NCI) Survey of Prostate Cancer in Accra^39^. To represent European ancestry, we used data from the CEU and IBS samples from the 1000 Genomes Project^19^. To represent Native American ancestry, we used data from Quechuas, Ashaninkas, Shimaas, and Aymaras (Tarazona-Santos Laboratory), Matsiguengas, Queros, Uros, and Moches^40^. To run RFMix, we set the number of generations since the admixture event (parameter -G) at 20 (~500 years) and the number of trees to generate per random forest (parameter -t) at 500. Inferences were performed in window lengths (parameter -w) of 0.2 cM. All other parameters in RFMix were set at default values. We considered only the windows in which ancestry was inferred with a posterior probability >0.90. The PCAdmix inferences and methods were performed as described elsewhere^11^.

### Admixture mapping

Local ancestry inferences were used to perform admixture mapping across the genome. Specifically, we tested for an association between the ACD as a continuous variable and the number of African, European, and Native American chromosomes (0, 1 or 2) at a locus. We used the linear mixed model implemented in GCTA^41^, adjusted for age, sex, schooling, and global African and Native American proportions as fixed effects. Furthermore, we used the kinship matrix estimated by REAP as a random effect.

To establish a significance threshold for admixture mapping, accounting both for multiple testing and the correlation of local ancestry between loci, we estimated the effective number of tests for each chromosome for each individual using autocorrelation^42^. We then used the effective number of tests in a partial Bonferroni correction; the significance threshold was 0.05 divided by the effective number of tests. We performed fine-mapping analysis for the genomic regions significantly associated in the admixture mapping, using both genotyped and imputed SNPs. We used the imputed SNPs ± 1 Mb from the midpoint of significant admixture mapping regions.

### Genome-wide association analysis (GWAS)

We performed genome-wide association analysis between each genotyped and imputed SNP and the trajectory of cognitive function using the linear mixed model implemented in GCTA^41^. The multiple linear regression models were adjusted for age, sex, schooling and the global African and Native American proportions as fixed effects. We used the kinship matrix estimated by REAP as a random effect.

### Significant SNPs and variance explained

To estimate the coefficient of determination (*R*^2^) from significant SNPs and the trajectory of cognitive function, we first selected the top associated SNPs one-by-one iteratively via a stepwise model selection procedure using the MASS library in R^43^ and then calculated the R^2^ of the final model. SNP heritability was calculated using REML analysis in the GCTA program^41^.

### Annotation and reproducibility

To annotate associated genetic variants, we used ANNOVAR^44^ and a set of web genomic resources^45–47^. Ancestry inferences, imputation, and fine-mapping were performed using the master scripts available in the EPIGEN-Brazil Scientific Workflow website (http://www.ldgh.com.br/scientificworkflow/index.html,)^48^. Admixture mapping pipelines of this manuscript are those developed for the admixture mapping of BMI in the three EPIGEN-Brazil Brazilian cohorts by Scliar *et al*.^49^.

### Ethics statement

The institutional review board of the Oswaldo Cruz Foundation, Rio de Janeiro, Brazil, fully approved the Bambuí Cohort Study of Aging. Brazil’s national research ethics committee approved genotyping as part of the Epigen-Brazil protocol (Brazilian National Ethics Research Council (CONEP), resolution 15895). All methods were performed in accordance with the CONEP guidelines and regulations. Written informed consent was obtained from all participants at baseline and at all follow-up interviews.

## Supplementary information

Table S1

Table S2

## Data Availability

EPIGEN-Brazil data are deposited in the European Nucleotide Archive (PRJEB9080 (ERP010139), Accession No. EGAS00001001245, under EPIGEN Committee Controlled Access mode. The Kwa/Gur datasets are deposited in dbGaP at phs000838.v1.p1. Botswana samples were obtained from dbGaP (phs001396.v1.p1).
