# Supplementary material for: Genetics of cognitive trajectory in Brazilians: 15 years of follow-up from the Bambuí-Epigen Cohort Study of Aging
Source: Sci Rep. 2019 Dec 2;9:18085. doi: 10.1038/s41598-019-53988-4 (PMC6889148; doi:10.1038/s41598-019-53988-4)
Supplement: Supplementary file 1 — Table S1 [file 41598_2019_53988_MOESM1_ESM.pdf]

Genetics of cognitive trajectory in Brazilians: 15 years of follow-up from the Bambuí-Epigen Cohort Study of Aging

Mateus H. Gouveia[*1,2,3], Cibele C. Cesar[4], Meddly L. Santolalla[2], Hanaisa P. Sant Anna[2], Marilia O. Scliar[2], Thiago P. Leal[2], Nathalia M. Araújo[2], Giordano B. Soares-Souza[2], Wagner C. S. Magalhães[2], Ignacio F. Mata[5], Cleusa P. Ferri[6], Erico Castro-Costa[1], Sam M. Mbulaiteye[7], Sarah A. Tishkoff[8], Daniel Shriner[3], Charles N. Rotimi[3] , Eduardo Tarazona-Santos[2], Maria Fernanda Lima-Costa[*1].

Table S1. Top results of the genome-wide association analysis (GWAS) of age-related cognitive decline in Bambuí-Epigen Cohort Study of Aging.

Chr	SNP	Allele	MAF	*b*	*p*-value	Mapped Genes
1	rs138347004	A/T	0.024678	-0.04441	3.33E-07	OTUD3, PLA2G2E
3	rs17008691	G/A	0.025942	-0.04264	6.21E-07	ZNF385D
3	rs78388807	G/A	0.025942	-0.04264	6.21E-07	ZNF385D
3	rs116488396	A/C	0.02596	-0.04264	6.22E-07	ZNF385D
3	rs4858320	G/A	0.025942	-0.04264	6.21E-07	ZNF385D
3	rs4857998	T/A	0.025942	-0.04264	6.21E-07	ZNF385D
3	rs115984210	C/T	0.025942	-0.04264	6.21E-07	ZNF385D
3	rs114613933	A/C	0.025942	-0.04264	6.21E-07	ZNF385D
3	rs75144331	A/G	0.025942	-0.04264	6.21E-07	ZNF385D
3	rs147517472	A/-	0.02596	-0.04274	5.86E-07	ZNF385D
3	rs76238412	G/A	0.02596	-0.04274	5.86E-07	ZNF385D
3	rs74584681	C/A	0.026297	-0.04385	2.47E-07	ZNF385D
3	rs79381650	C/T	0.026297	-0.04385	2.47E-07	ZNF385D
6	rs190722109	G/A	0.011396	-0.06822	1.24E-07	RP1-149C7.1, AL359987.1
6	rs145261073	A/G	0.012626	-0.06069	8.73E-07	RP1-149C7.1, AL359987.2
7	rs78400729	A/G	0.014235	-0.05813	3.47E-07	RP11-122G11.1, VWC6
7	rs150356279	A/-	0.014215	-0.05816	3.42E-07	RP11-122G11.1, VWC2
7	rs75968745	G/A	0.014215	-0.05816	3.42E-07	RP11-122G11.1, VWC3
7	rs17133460	T/C	0.014215	-0.05816	3.42E-07	RP11-122G11.1, VWC4
7	rs79868020	C/T	0.014215	-0.05816	3.42E-07	RP11-122G11.1, VWC5
7	rs77499314	G/A	0.010957	-0.0678	8.52E-08	RP11-807H17.1, AC005276.1
14	rs117922293	A/G	0.027719	-0.04239	3.21E-07	NRXN3
14	rs79654683	A/G	0.026372	-0.04165	9.48E-07	NRXN4
14	rs117556367	T/G	0.026372	-0.04165	9.48E-07	NRXN5
