# Supplementary material for: Genetics of cognitive trajectory in Brazilians: 15 years of follow-up from the Bambuí-Epigen Cohort Study of Aging
Source: Sci Rep. 2019 Dec 2;9:18085. doi: 10.1038/s41598-019-53988-4 (PMC6889148; doi:10.1038/s41598-019-53988-4)
Supplement: Supplementary file 2 — Table S2 [file 41598_2019_53988_MOESM2_ESM.pdf]

Genetics of cognitive trajectory in Brazilians: 15 years of follow-up from the Bambuí-Epigen Cohort Study of Aging

Mateus H. Gouveia[*1,2,3], Cibele C. Cesar[4], Meddly L. Santolalla[2], Hanaisa P. Sant Anna[2], Marilia O. Scliar[2], Thiago P. Leal[2], Nathalia M. Araújo[2], Giordano B. Soares-Souza[2], Wagner C. S. Magalhães[2], Ignacio F. Mata[5], Cleusa P. Ferri[6], Erico Castro-Costa[1], Sam M. Mbulaiteye[7], Sarah A. Tishkoff[8], Daniel Shriner[3], Charles N. Rotimi[3], Eduardo Tarazona-Santos[2], Maria Fernanda Lima-Costa[*1].

Table S2. Association analysis for age-related cognitive decline trajectory (Bambuí-Epigen Cohort Study of Aging) using the GWAS catalog SNPs searched with terms "cognitive decline" and "Alzheimer disease".

Chr	SNP	MAF	b	p-value	PUBMED ID	REGION	MAPPED GENES	Risk Allele
19	rs429358	0.139653	-0.01551	5.99E-05	23419831	19q13.32	APOE	C
19	rs157582	0.2715	-0.00962	0.001385	26421299	19q13.32	TOMM40	NR/T
19	rs115881343	0.021521	-0.02758	0.002773	24468470	19q13.32	TOMM40	NR/T
19	rs6857	0.120172	-0.0123	0.003099	28183528	19q13.32	PVRL2	T
19	rs483082	0.224659	-0.00958	0.003182	28183528	19q13.32	APOE,APOC1	T
19	rs283811	0.230798	-0.00969	0.003261	28183528	19q13.32	PVRL2	G
19	rs4420638	0.159204	-0.01073	0.003719	26830138	19q13.32	APOC1,APOC1P1	A
19	rs56131196	0.157783	-0.01074	0.003747	23419831	19q13.32	APOC1,APOC1P1	A
2	rs1443024	0.3125	-0.00851	0.003889	26830138	2q32.1	LOC105373779,LOC105373780	A
19	rs769449	0.094172	-0.01213	0.008757	23562540	19q13.32	APOE	A
5	rs71636213	0.06066	-0.01459	0.009869	25778476	5q14.1	SERINC5,KRT18P45	G
20	rs34972666	0.126155	-0.01023	0.013228	23535033	20p13	TGM6	NR/G
19	rs59007384	0.240807	-0.0078	0.014258	23419831	19q13.32	TOMM40	T
4	rs77803164	0.047974	-0.01513	0.015908	24468470	4q31.1	LOC105377448	NR/A
19	rs394819	0.015636	-0.02328	0.021694	26339675	19q13.32	TOMM40	T
8	rs150639459	0.012563	-0.02779	0.021917	26252872	8p22	PSD3	C
1	rs79036927	0.03257	-0.01698	0.022541	26830138	1q23.3	LOC105371521,NMNAT1P2	A
17	rs149464898	0.011111	-0.0265	0.023903	26830138	17q25.3	LOC400627,RPL12P37	T
13	rs9564655	0.1267	-0.00877	0.029318	28577822	13q21.33	ATXN8OS	NR/T
12	rs11610206	0.078891	0.010682	0.030269	19118814	12q13.11	LOC105369746	NR/C
5	rs17716202	0.032547	-0.01635	0.032157	22005930	5q11.2	LOC101928448,LOC105378979	NR/C
2	rs12468965	0.023099	0.018694	0.032964	26252872	2p16.2	LOC105369165,RNU6-997P	G
7	rs802571	0.157072	-0.00781	0.034015	26049409	7q35	CNTNAP2	T
3	rs10937470	0.422581	-0.00569	0.035286	21116278	3q28	UTS2B	NR/T
17	rs71370433	0.020563	-0.01914	0.045409	26252872	17p13.2	RNU6-1264P,BTF3P14	C
8	rs76070545	0.024731	-0.01744	0.046828	26830138	8q22.3	FZD6,CTHRC1	T

NR/allele = Non-reported risk allele/minor allele
